# Breast acinic cell carcinoma with weak progesterone receptor expression: a case report and literature review

**DOI:** 10.3389/fonc.2024.1497272

**Published:** 2025-02-20

**Authors:** Caiyun Bai, Xiaodong Xin, Yisen Yang, Fengjiang Qu, Zhimin Fan

**Affiliations:** ^1^ Breast Surgery Department, General Surgery Center, The First Hospital of Jilin University, Changchun, China; ^2^ Emergency Surgery Department, The First Hospital of Jilin University, Changchun, China

**Keywords:** breast cancer, acinic cell carcinoma, progesterone receptor, adjuvant chemotherapy, microglandular adenosis

## Abstract

**Rationale:**

Acinic cell carcinoma (AcCC) of the breast is an extremely rare malignant epithelial tumor characterized by acini cell differentiation, clinical low-grade malignancy, and a molecular triple-negative subtype.

**Patient concern:**

A 47-year-old female presented with a 1-month history of a painless mass in her right breast.

**Diagnosis:**

Ultrasound imaging, mammography and magnetic resonance imaging revealed a lesion, approximately 3.0cm×1.5cm in size, in the right breast, which was considered to be a malignancy. After the surgery, the AcCC of the breast was confirmed histologically.

**Interventions:**

Right breast mastectomy and sentinel lymph node biopsy were performed. Adjuvant chemotherapy included 4 cycles of doxorubicin hydrochloride (Adriamycin) and cyclophosphamide followed by 4 cycles of docetaxel (Taxotere).

**Outcomes:**

The patient was discharged from the hospital after surgery. There was no sign of recurrence during a 9-month follow-up period.

**Lessons:**

Acinic cell carcinoma (AcCC) of the breast is an extremely rare malignant epithelial tumor that can be accurately diagnosed based on histopathologic morphology and immunohistochemistry. The weak positive progesterone receptor (PR) expressed in this case is extremely rare, which may provide a new research direction for the endocrine therapy of AcCC. Both AcCC and microglandular adenosis(MGA) exhibit microglandular growth, and the relationship between them remains unclear. Differentiation between them not only relies on histomorphology and pathological immunohistochemistry but also depends on clinical manifestations and other presentations. Optimal treatment of AcCC is the same as that for invasive breast cancer. The prognosis is generally good, with adjuvant therapy after surgery.

## Introduction

Breast cancer, is a heterogeneous disease, originating from the breast epithelial cells, and it falls under the category of adenocarcinomas. In the latest WHO classification, breast cancer is divided into 18 types, including invasive ductal carcinomas of no special type and 17 special types. Pathologists have identified specific structures and cytological patterns that align with certain clinical presentations and/or outcomes, termed “histological special types,” which account for 25% of all breast cancers, include micropapillary carcinoma, apocrine carcinoma, metaplastic carcinoma, medullary carcinoma, adenoid cystic carcinoma, secretory carcinoma, acinic cell carcinoma, and so on. Special types of breast cancer hold significant clinical importance due to their unique biological characteristics. However, there is a lack of standardized diagnostic criteria for these special types of breast cancer. Acinic Cell Carcinoma (AcCC) is an extremely rare special type of breast cancer characterized by acinic cell differentiation and low malignancy. Its molecular subtype is triple-negative (estrogen receptor-negative, progesterone receptor-negative, and Her-2 negative) predominantly, with immunohistochemical features often positive for S-100 and EMA, high expression of lysozyme, and stain positive for periodic acid-Schiff (PAS) with diastase (PAS-D). Here, we report the clinical case of a 47-year-old female patient with breast AcCC with weak progesterone receptor (PR) expression and review the relevant literature.

## Case information

**Figure 1:**
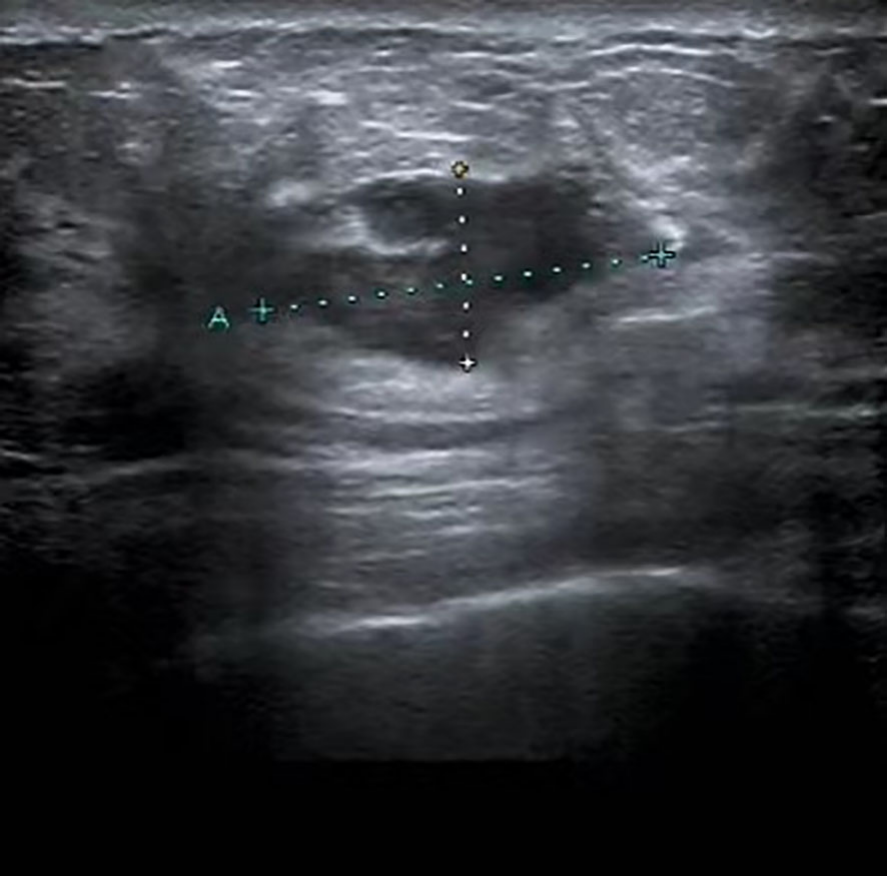
Ultrasound image of the breast: A mass, measuring 25.4 mm by 12.5 mm, was identified in the upper quadrant of the right breast, near the three o’clock position. This mass has indistinct margins and an irregular contour.

**Figure 2:**
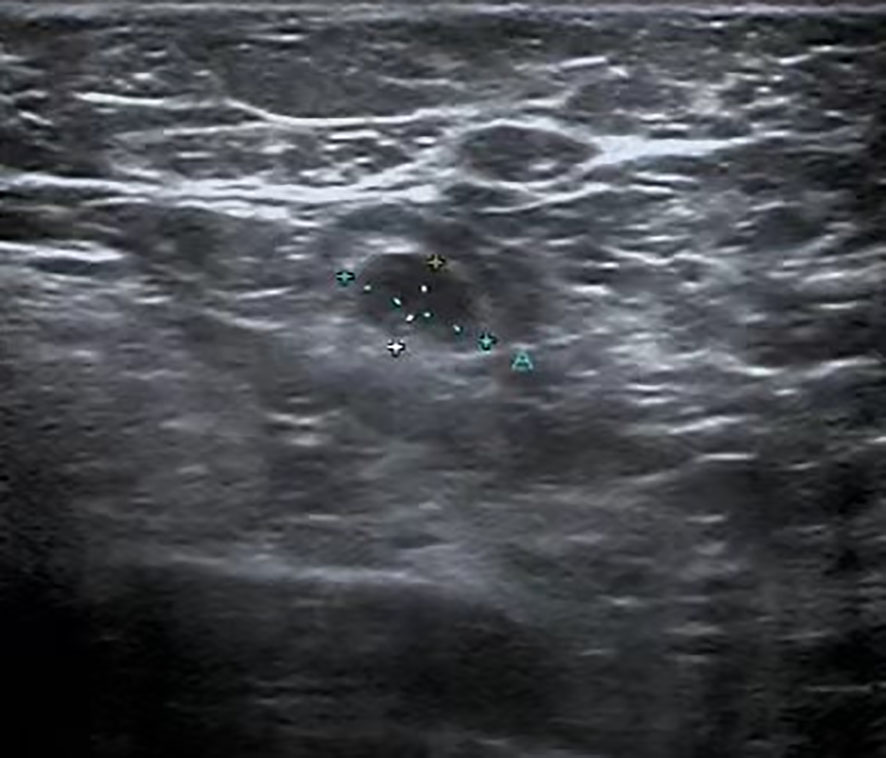
Ultrasound image of the axilla: The largest lymph node in the right axilla, measuring 9.7mm by 5.9mm, has lost its normal architecture.

**Figure 3:**
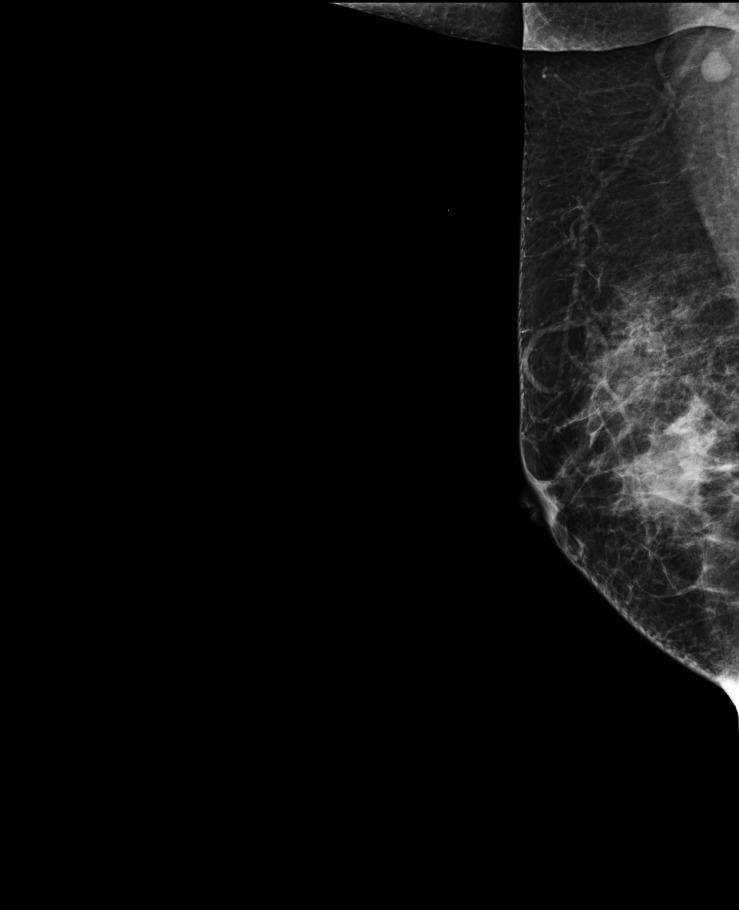
X-ray image of right breast mass and axillary lymph nodes: The structure of the local glands in the upper quadrant of the right breast was slightly disordered with increased density; lymph node shadows were seen in the right axilla with increased density.

**Figure 4:**
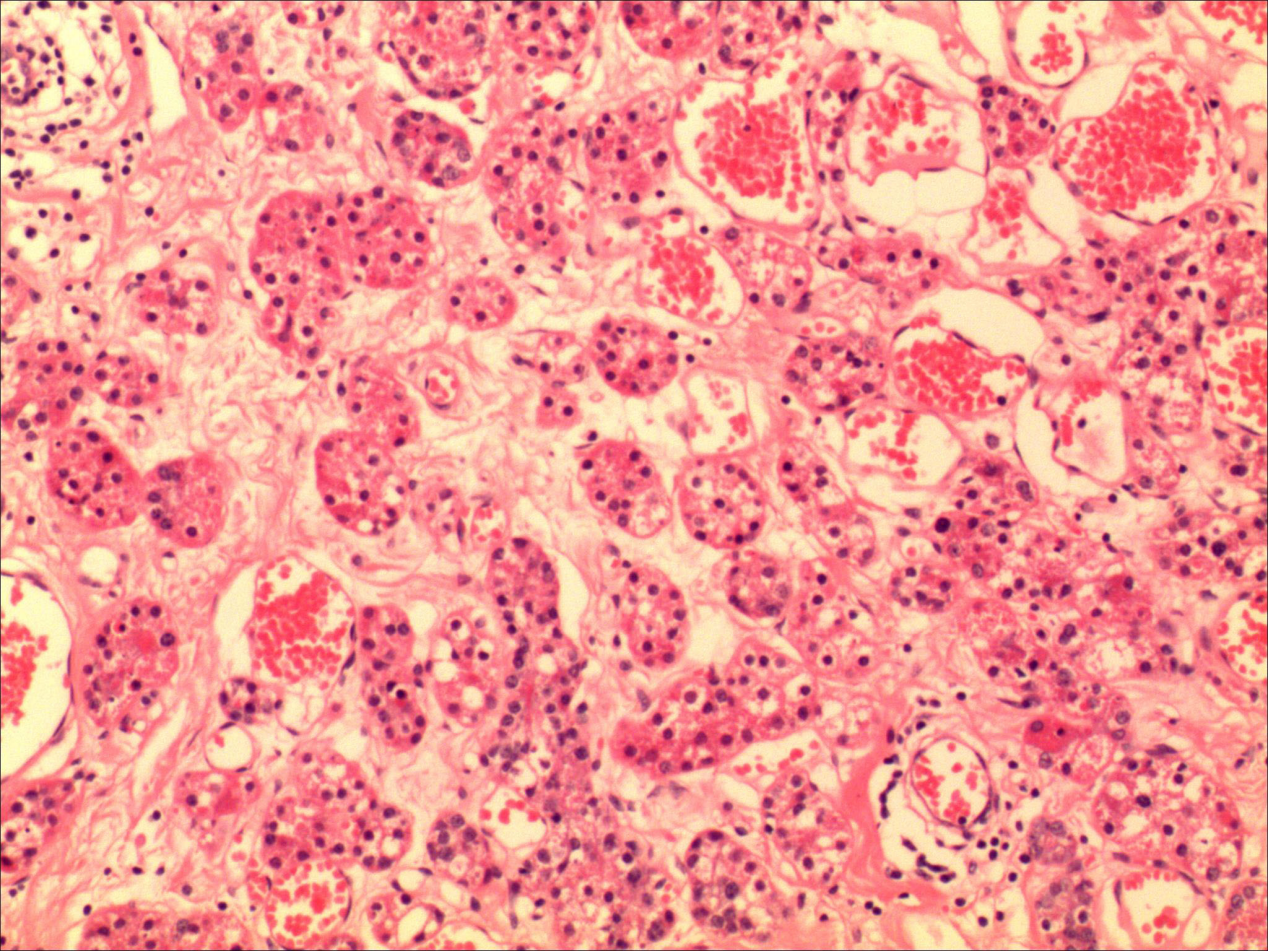
The pathological image shows acinar hyperplasia, with the acini being irregular and composed of multilayered cell arrangements.

A 47-year-old female presented with an accidental discovery of a broad bean-sized lump in her right breast in September 2021. The lump was painless, without nipple discharge or nipple retraction. She visited a local hospital and underwent a core needle biopsy, which suggested invasive carcinoma. She was then referred to our hospital on Nov 13, 2021, for further treatment. Physical examination: A round, hard mass approximately 3.0 cm × 1.5 cm in size was palpable in the upper inner quadrant of the right breast, with poorly defined borders, no orange peel-like changes, and no enlarged lymph nodes in the bilateral axilla and supraclavicular region. Imaging examination: Ultrasound of breast and axilla: A hypoechoic mass with a size of 25.4 mm × 12.5 mm was located near three o’clock position in the upper quadrant of the right breast, with unclear boundaries and irregular shape, with blood flow signals observed, indicative of a prompt right breast BI-RADS IVb tumor (**Figure 1**). Some lymph nodes in the right axilla had lost normal structure, with the largest 9.7mm × 5.9 mm (**Figure 2**). Mammography: The structure of the local glands in the upper quadrant of the right breast was slightly disordered with increased density BI-RADS 0; lymph node shadows were seen in the right axilla with increased density (**Figure 3**). Breast magnetic resonance imaging (MRI) plain scan + enhanced + diffusion: Irregular-shaped abnormal signal shadow in the upper quadrant of the right breast, approximately 2.7 × 2.2 cm, T1 fat-suppressed image showed iso-intensity, T2 fat-suppressed image showed slight hyper-intensity. Diffusion-weighted MRI (DWI) showed hyper-intensity. Antibody-drug conjugate (ADC) showed a low signal, lobulation, and burr at the edge of the lesion, with an uneven enhancement and a time-signal intensity curve exhibiting a rapid-rising type. The right breast tumor was classified as BI-RADS-MR V. With enhancement, a small lymph node shadow was seen in the right axilla of approximately 0.7 cm. Results did not rule out the possibility of axillary lymph node metastasis, and a right axillary lymph node biopsy was performed, and no cancer invasion was seen. There were no clear signs of metastasis on chest and abdomen computed tomography (CT) and whole-body bone imaging, with no surgical contraindications. On Nov 23, 2021, a right breast mastectomy and sentinel lymph node biopsy were performed. Two sentinel lymph nodes were evaluated during the operation. No cancer metastasis was found. Postoperative pathology: breast acinic cell carcinoma (**Figure 4**), volume 2.3 cm × 2.0 cm × 1.5 cm, with no cancer invasion in vessels, nerves, superficial fascia, or breast tissue in the remaining quadrant. Immunohistochemistry (**Supplementary Figures 1, 2**): PR (weak + 20%) (**Supplementary Figure 3**), ER (-) (**Supplementary Figure 4**), Her-2 (0) (**Supplementary Figure 5**), CK7 (+), GATA3 (+), GCDFP-15 (+), EMA (+), S-100 (+), CK5/6 (partly +), p63 (-), Ki-67 (+70%) (**Supplementary Figure 6**), Syn (-), CgA (-), AR (-), ECadherin (+) (**Supplementary Figure 7**), lysozyme (+) (**Supplementary Figure 8**), and special stain: D-PAS (+). Currently, the patient has successfully undergone eight cycles of AC-T adjuvant chemotherapy, 4 cycles of doxorubicin hydrochloride (Adriamycin) and cyclophosphamide followed by 4 cycles of docetaxel (Taxotere). Her condition remains stable, and there have been no indications of tumor recurrence observed during subsequent follow-ups.

## Discussion

Breast cancer is a heterogeneous disease, originating from malignant tumors of breast epithelial cells, and it belongs to adenocarcinomas. In the latest WHO classification, breast cancer is divided into 18 types, including invasive ductal carcinomas of no special type (IDC-NST) and 17 special types. Special types of breast cancer account for 25% of all breast cancers (1), include micropapillary carcinoma, apocrine carcinoma, metaplastic carcinoma, medullary carcinoma, adenoid cystic carcinoma, secretory carcinoma, acinic cell carcinoma, and so on (1, 2). Pathologists have identified that over 90% of tumor entities with recognized special type morphological characteristics are pure special types. Special types of breast cancer are of significant clinical importance due to their unique biological features. However, there is a lack of standardized diagnostic criteria for special types of breast cancer due to the scarcity of reports and low interobserver reproducibility (3).

AcCC is a rare malignant epithelial tumor that belongs to salivary gland-like carcinoma, which is a special type of invasive cancer (4, 5). AcCC is a low-grade malignant salivary gland epithelial tumor with serous acinar cell differentiation, accounting for 17% of salivary gland tumors (6). The parotid gland is the primary site of origin, with approximately 2%-3% of parotid tumors being primary adenoid cystic carcinomas (7). It was first breast AcCC reported by Roncaroli in 1996 (8), and most of the literature since then has been case reports. We found 52 case reports in English on PubMed, including the only male breast AcCC reported by Shimao et al. (9). According to a recent report, The mean patient age at diagnosis was 51 years (median age, 49; range, 23–80 years), with a higher incidence on the right breast, though no specific quadrant preference has been observed, as evidenced in this case (4). Physical examination and imaging showed Nodules with unclear borders, irregular morphology, and slightly hard texture (10, 11).In addition, axillary metastasis is rare in AcCC cases, and the pathological results of our patient did not show axillary metastasis (4, 12). There is no characteristic description of AcCC in the existing literature resembling those of breast cancer, with irregular morphology, poorly defined borders, visible blood flow signals, and nonhomogeneous enhancement in enhanced MR, so the accurate diagnosis relies on pathological examination.

Breast tissue shares histomorphology and functional similarities with most secretory glands, e.g., the salivary glands and the mammary glands are composed of tubular alveoli with the same histological structure (4). It is, therefore, not surprising that breast tumors present as salivary gland-like carcinomas, including acinic cell carcinoma, polymorphous adenocarcinoma, mucoepidermoid carcinoma, secretory carcinoma and adenoid cystic carcinoma (5, 13). Genomic abnormalities in some types of primary salivary gland tumors can also be detected in salivary gland-like primary tumors of the breast (e.g., polymorphous adenocarcinoma with HMGA2 or PLAG1 rearrangement; Secretory carcinoma with T (12; 15) fusion with ETV6-NTRK3; or adenoid cystic carcinoma with t(6; 9), resulting in MYB-NFIB fusion) (14).AcCC commonly occurs in salivary glands and resembles salivary gland acinic cell carcinoma morphologically when found in the breast. AcCC of the salivary glands exhibits a distinct morphological presentation, characterized by well-defined tumor margins, a prominent capsule, and a variety of morphological growth patterns (15). Typical breast AcCC shows adenoalveolar cell differentiation with cytoplasm enriched with enzyme-producing granules (4). The cytoplasm is mostly eosinophilic but can also be basophilic or a mixture of eosinophilic and basophilic (16). However, breast AcCC is still somewhat different from salivary gland AcCC, with smaller borders and less invasion compared to salivary gland AcCC (13, 17). Compared with salivary gland AcCC, breast AcCC is more similar to triple-negative breast cancer (TNBC) with no special types. For example, mutation of TP53, which is the most common mutation pattern in breast cancer, was found in 80% of AcCC cases, but this mutation was not detected in salivary gland AcCC (18); however, AcCC aggressiveness is far less than triple-negative breast cancer (4); in terms of clinical manifestations and morbidity are also quite different (5).

AcCC exhibits various growth patterns, among which the cells arranged in solid with or without microglandular growth pattern are predominant, and which is one of the characteristics of microglandular adenosis (MGA) of the breast (4). AcCC of the breast can show local morphological features similar to those of MGA; the presence of rare, rough, bright eosinophilic cytoplasmic granules in the breast epithelium is a characteristic feature of the tumor, mainly found in MGA lesions and AcCC (16). Therefore, the cytological and histological features are particularly important in the diagnosis of AcCC.MGA is a benign breast lesion, and 4 cases of breast AcCC have been reported to occur on the basis of MGA (10–13), while the real relationship between these two diseases remains unclear (16). Scholars hold diverse opinions on the relationship between them. With Rosen suggesting that breast AcCC is invasive carcinoma with acinic cell differentiation arising in microglandular adenosis (19), Geyer et al. pointed out that MGA and AcCC represent the low-grade spectrum of relatively inert TNBC lesions and share some common molecular features but that they are two distinct lesions (20); and Conlon N advocated that AcCC occurs in the stroma of MGA, and the resection margin should be re-examined if MGA lesions are present when breast-conserving surgery is performed for AcCC (16). However, this phenomenon is not absolute in rare case reports, so the differentiation between the two depends not only on histomorphology and pathological immunohistochemistry but also on clinical and other presentations.

Detailed descriptions of immunohistochemical characterization of breast AcCC have been reported in some of the breast AcCC literature, and the classical immunohistochemical features of breast AcCC: high expression of S-100, EMA, lysozyme and positive for PAS (21). The basic manifestations of AcCC reported in the existing literature are triple negative. AcCC belongs to the triple-negative breast cancer (TNBC) subtype; the number of AcCC that do not show triple negative is less than about 10% (4, 16). S-100 and EMA positivity are the main markers for distinguishing AcCC from serous carcinoma; typical breast AcCC shows acinar cell differentiation and lysozyme-positive cytoplasm rich in enzyme-producing granules, which are positive to the special staining PAS and PAS-D (4). The testing for the myoepithelial marker p63 was negative, confirming the invasive nature of this tumor type (21). As with other epithelial tumors, AcCC was positive for broad-spectrum cytokeratins and low molecular weight cytokeratins (e.g., CK7 and CK18). As with no specific type of triple-negative breast cancer, high molecular weight cytokeratins (e.g., CK5/6) can also be detected. Proliferation rates, as measured by Ki-67, show a wide positive range (5-71%); however, the majority of studies have reported that AcCC exhibits low to moderate Ki67 expression (range 5-30%) (4). In addition, GATA3 and GCDFP-15, which are apocrine differentiation markers, are expressed in about 1/2 of the AcCC cases. Furthermore, there are cases showing mutations in CTNNB1 (encoding β-adhesin), but no mutations in CDH1 (encoding E-Cadherin) have been identified (22, 23). The case we report in this article shows a general agreement with the above description. The special point is that the progesterone receptor (PR) is weakly positive and shows positive for E-cadherin, though no genetic testing has been performed, and our case also showed 70% of ki- 67 high expression.Due to the rarity of AcCC and the limited number of reported cases, there is a lack of clear diagnostic criteria for AcCC, both in salivary gland cancers and breast cancers. A meta-analysis has indicated that among 333 salivary gland AcCC patients, a DOG1 expression rate of 55% was observed, suggesting a strong correlation between the expression of this marker and the final diagnosis of AcCC. So DOG1 can serve as an immunohistochemical marker for salivary gland AcCC (24), and pathological evidence along with immunohistochemistry can provide a diagnostic basis for breast AcCC.

With advancements in molecular biology and immunohistochemistry, there is increasing evidence to support the presence of individuals with the ER-negative/PR-positive subtype, which represents a very small minority and is more commonly found in younger patients (<49 years of age). The prognosis of ER-negative/PR-positive is worse than that of ER-positive/PR-positive or ER-positive/PR-negative breast cancers and is superior to that of ER-negative/PR-negative breast cancers (16). 31% of ER-negative/PR-positive patients are ERBB2-positive, which is not significantly different from the ERBB2 status between ER-negative/PR-negative subtypes (25.3% vs 29.5%). Some researchers have also suggested that ER-negative/PR positivity is due to inadequate tissue fixation or failure of immunohistochemical detection technology in ER-positive/PR-positive breast cancers (25–27). However, with the optimization of immunohistochemical technology, there has been a decrease in ER-negative/PR-positive breast cancers. Conlon also reported a case of ER-negative/PR-positive AcCC (16).

The PR-RANK-RANKL pathway is responsible for progesterone-mediated abnormal proliferation of breast epithelial cells (28). It has been shown that inhibition of RANKL directly leads to a reduction in early hormone-induced breast epithelial tumourigenesis (29). PR is involved in molecular subtypes and plays a decisive factor in therapeutic decisions. However, its complex role in different contexts needs to be specifically elucidated. The growth of breast cancer cells that express positive for ER and/or PR depends on estrogen and/or progesterone. The expression of ER and/or PR is a key determinant of whether patients can benefit from endocrine therapy targeting the effects of estrogen, such as selective ER modulators (SERMs, e.g., tamoxifen), aromatase inhibitors (letrozole, anastrozole, exemestane), and estrogen receptor downregulation (fulvestrant). The WHO defines luminal A breast cancer as hormone receptor (HR) positive, HER2 negative, with Ki-67 less than 14% and PR greater than 20%, while luminal B is defined as estrogen receptor (ER) positive, HER2 negative, with Ki-67 greater than 14% or PR less than 20%. PR is an independent prognostic factor for breast cancer, and even though clinically, PR-positive breast cancer consistently responds better to endocrine therapy than PR-negative breast cancer (30), the poor prognosis of PR-negative breast cancer may be related to endocrine resistance (28). However, PR expression is not yet predictive of the efficacy of endocrine drugs in clinical trials and may be related to the fact that the ER+/PR- genome has the same gene expression pattern as the ER+/PR+ and ER-/PR- genomes (28). Currently, the combined use of ovarian function suppression (OFS) with hormone receptor inhibitors has also led to breakthroughs in endocrine therapy. In the subgroup with PR expression of 20-49%, exemestane plus OFS showed a more favorable disease-free survival (DFS) compared to tamoxifen plus OFS, with HR= 0.43. Similar trends were observed in the PR<20% and >50% subgroups, with HRs of 0.59 and 0.75, respectively. Therefore, upgrading endocrine therapy or switching to alternative endocrine drugs may overcome endocrine resistance.

Generally speaking, TNBC is more aggressive and has a poorer prognosis compared to Her2-overexpressing and hormone receptor-positive breast cancers. Due to the diversity in tumor histological histology, TNBC encompasses a range of malignant tumors with varying clinical behaviors. Similarly, the lack of HER-2 expression is categorized into HER-2 low expression and ultra-low expression (31). Histologically distinct subtypes of TNBC, such as adenoid cystic carcinoma, medullary carcinoma, and apocrine carcinoma, tend to have a better prognosis than other types of invasive ductal carcinomas (32, 33). However, there are fewer reports on AcCC of the breast (2, 34). Recently, there have been reports of AcCC with poor prognosis, including cases with distant metastasis to the lungs, bones, liver, and even the peritoneum (1, 10). Among the 68 cases reported in the literature, four had local metastasis, six developed distant metastasis after adjuvant therapy, and three died due to tumor progression (4, 30). Given the morphological variations within and between tumors (pure and mixed types) in the published studies, it is challenging to draw definitive conclusions about the prognosis of breast AcCC from the existing literature. Some experts believe that low-grade pure AcCC is a more indolent tumor type, similar to MGA esions, and is associated with an indolent biological behavior, thus less likely to benefit from aggressive adjuvant chemotherapy and has a better prognosis. In contrast, high-grade AcCC or AcCC mixed with other types of breast cancer may exhibit more aggressive behavior (35). However, due to the limited number of AcCC cases reported and the lack of observer reproducibility, further research is needed to identify specific high-risk factors for poor prognosis.

Treatment for AcCC is similar to that for invasive breast cancer, and surgery can be breast-conserving or mastectomy, based on tumor characteristics and patient preference. Most patients receive chemotherapy, and hormone receptor-positive cases undergo endocrine therapy. Some patients also received neoadjuvant therapy for breast cancer, but neoadjuvant therapy is not the first choice for AcCC treatment due to the relative inertia nature of AcCC and the relatively low ki-67, which makes neoadjuvant therapy ineffective (4). Curigliano’s study showed that TNBC subtypes are highly sensitive to platinum salts and taxanes (36). This patient received AC-T adjuvant chemotherapy after surgery and completed all cycles with no signs of local recurrence or distant metastasis. Due to the rarity of AcCC, evidence for its molecular genomics is scarce, and targeted therapies against specific signaling pathway targets could be used in the future when sufficient data are available. The use of immunosuppressive agents could also serve as a reference for individualized treatment.

## Conclusion

AcCC of the breast is a rare malignant tumor of the breast; due to its rarity, there is still limited evidence on its molecular genomics, making pathological evidence and the immunohistochemical analysis extremely valuable in this disease. Accurate diagnosis of AcCC can be made on the basis of pathohistological morphology and especially immunohistochemical expression. In most cases, high expression of S-100, EMA, lysozyme, and positive for PAS is observed, and most of the cases showed triple negative. The weakly positive expression of (PR) in this case is extremely rare and is accompanied by positive E-cadherin expression. The cases in this article provide new evidence for the field of endocrine therapy of AcCC and are beneficial to its development. E-Cadherin, encoded by CDH1, suggests that it may serve as a novel signal in the research field of AcCC, providing a new target for targeted therapy. Unfortunately, no genetic testing was performed in this case. Both AcCC and MGA exhibit microglandular growth patterns, but the relationship between them remains unclear. The expression of cohesin and type IV collagen was different, with MGA positive, while the AcCC was the opposite. However, in rare cases, this phenomenon is not absolute, so the differentiation depends not only on histomorphology and pathological immunohistochemistry but also on clinical presentation and other manifestations. The treatment is the same as invasive breast cancer, and the prognosis is generally good, with further adjuvant treatment after surgery. With the gradual development of the breast cancer endocrine field, endocrine drugs can also be added according to the expression of PR.

## Data Availability

All data generated or analyzed during this study are included in this published article.

## Supplementary material

Supplementary Figure 1 Breast acinic cell carcinoma pathology (HE * 400).

Supplementary Figure 2 Breast acinic cell carcinoma pathology (HE * 400).

Supplementary Figure 3 Immunohistochemistry: Weak PR receptor expression.

Supplementary Figure 4 Immunohistochemistry: ER is negative.

Supplementary Figure 5 Immunohistochemistry: Her-2 is negative.

Supplementary Figure 6 Immunohistochemistry: Ki-67 expressed 70%.

Supplementary Figure 7 Immunohistochemistry: E-Cadherin is positive.

Supplementary Figure 8 Lysozyme is positive, which is a marker of serous differentiation.
