# Supplementary figures and images for: Breast acinic cell carcinoma with weak progesterone receptor expression: a case report and literature review
Source: Front Oncol. 2025 Feb 20;14:1497272. doi: 10.3389/fonc.2024.1497272 (PMC11883444; doi:10.3389/fonc.2024.1497272)

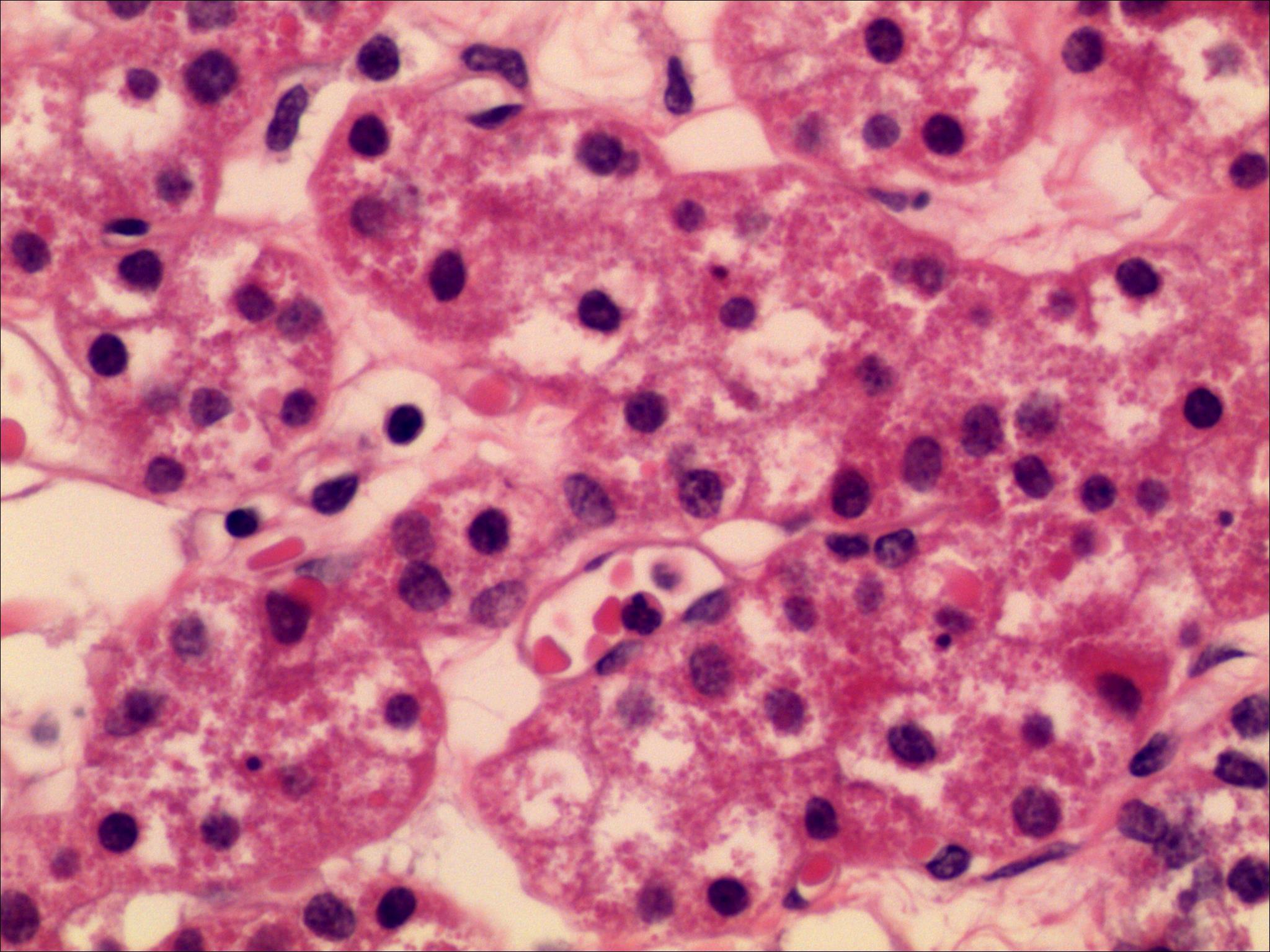

Supplement: Supplementary Figure 1 — Breast acinic cell carcinoma pathology (HE * 400). [file Image1.jpeg]

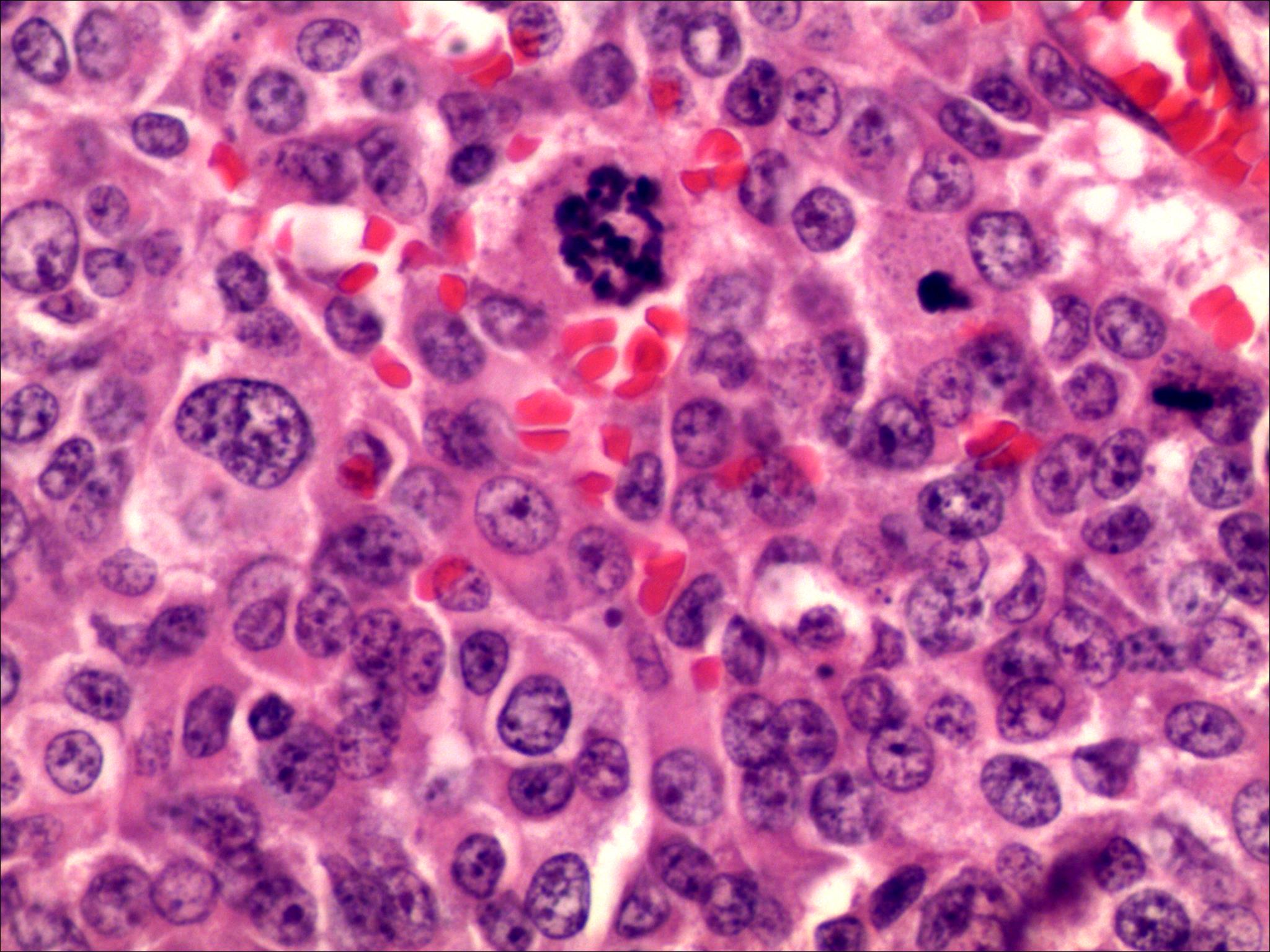

Supplement: Supplementary Figure 2 — Breast acinic cell carcinoma pathology (HE * 400). [file Image2.jpeg]

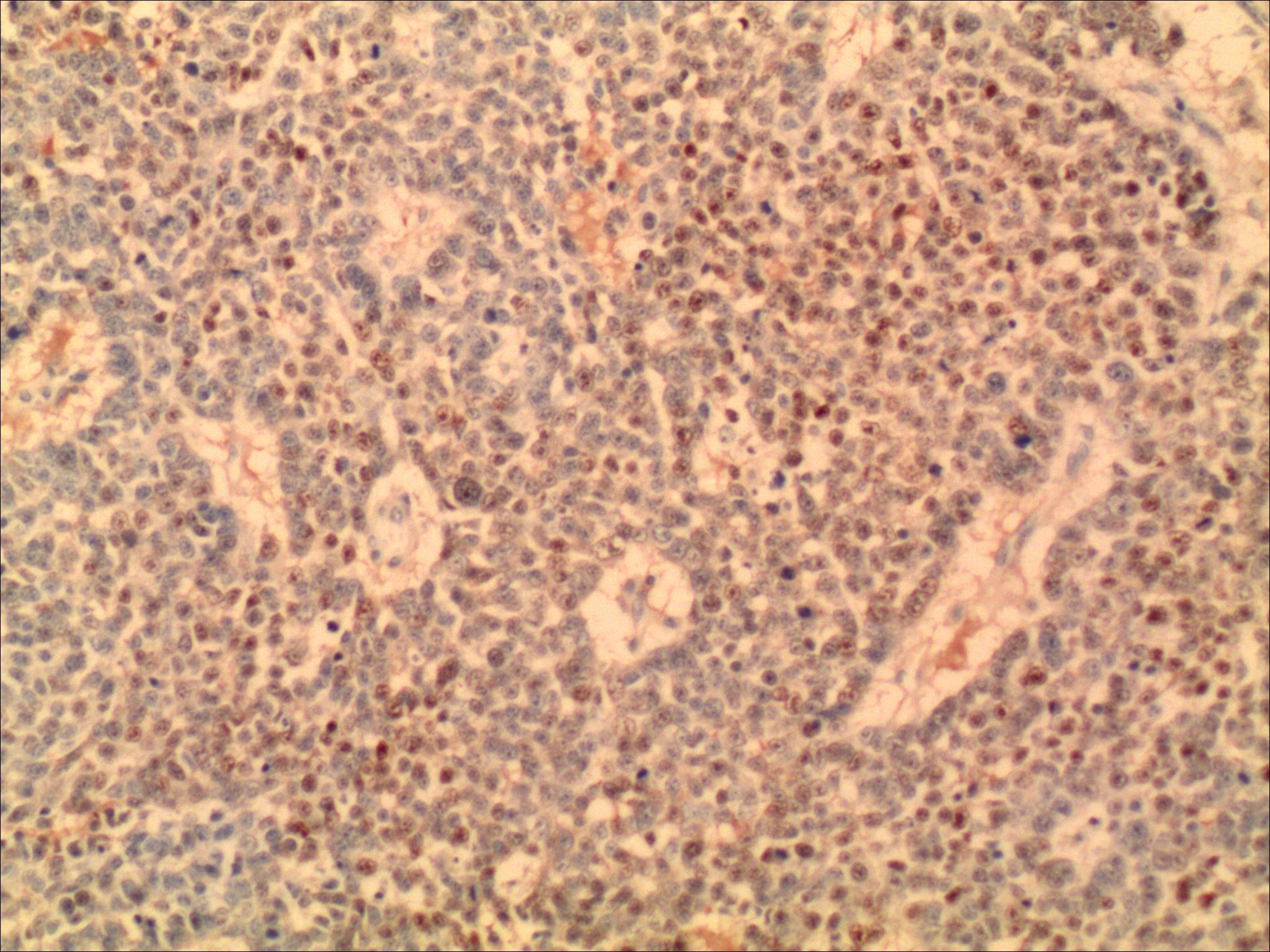

Supplement: Supplementary Figure 3 — Immunohistochemistry: Weak PR receptor expression. [file Image3.jpeg]

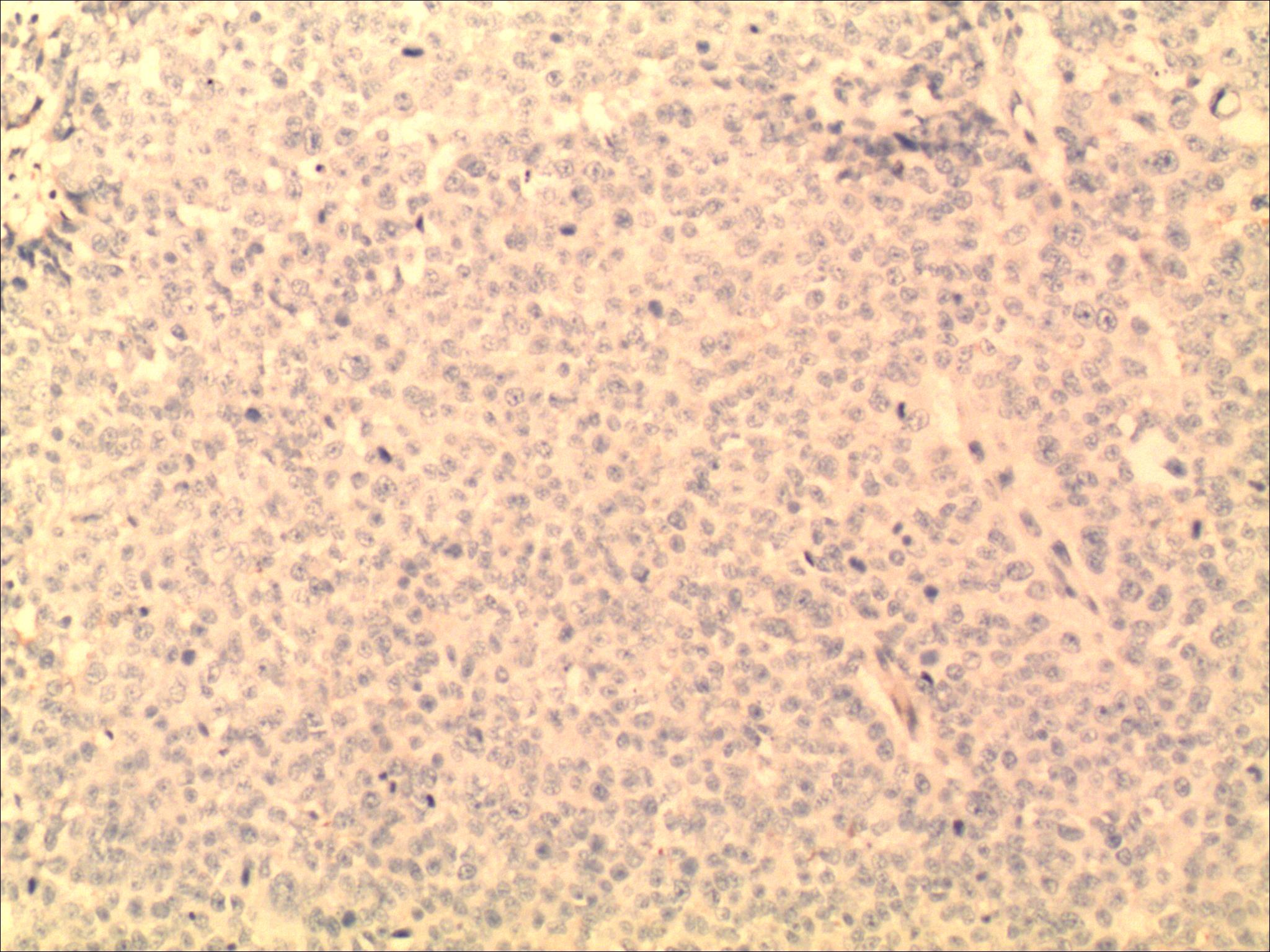

Supplement: Supplementary Figure 4 — Immunohistochemistry: ER is negative. [file Image4.jpeg]

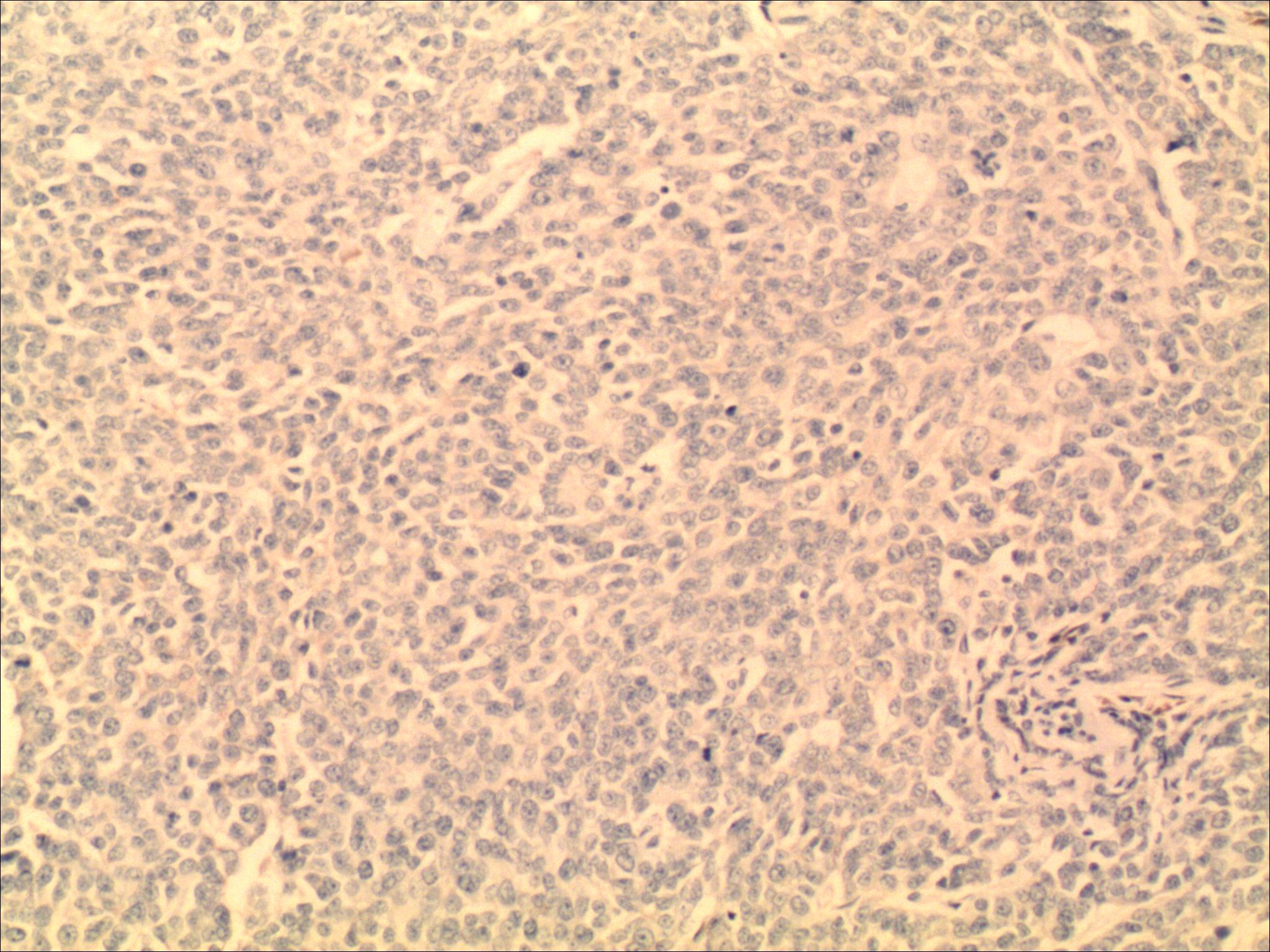

Supplement: Supplementary Figure 5 — Immunohistochemistry: Her-2 is negative. [file Image5.jpeg]

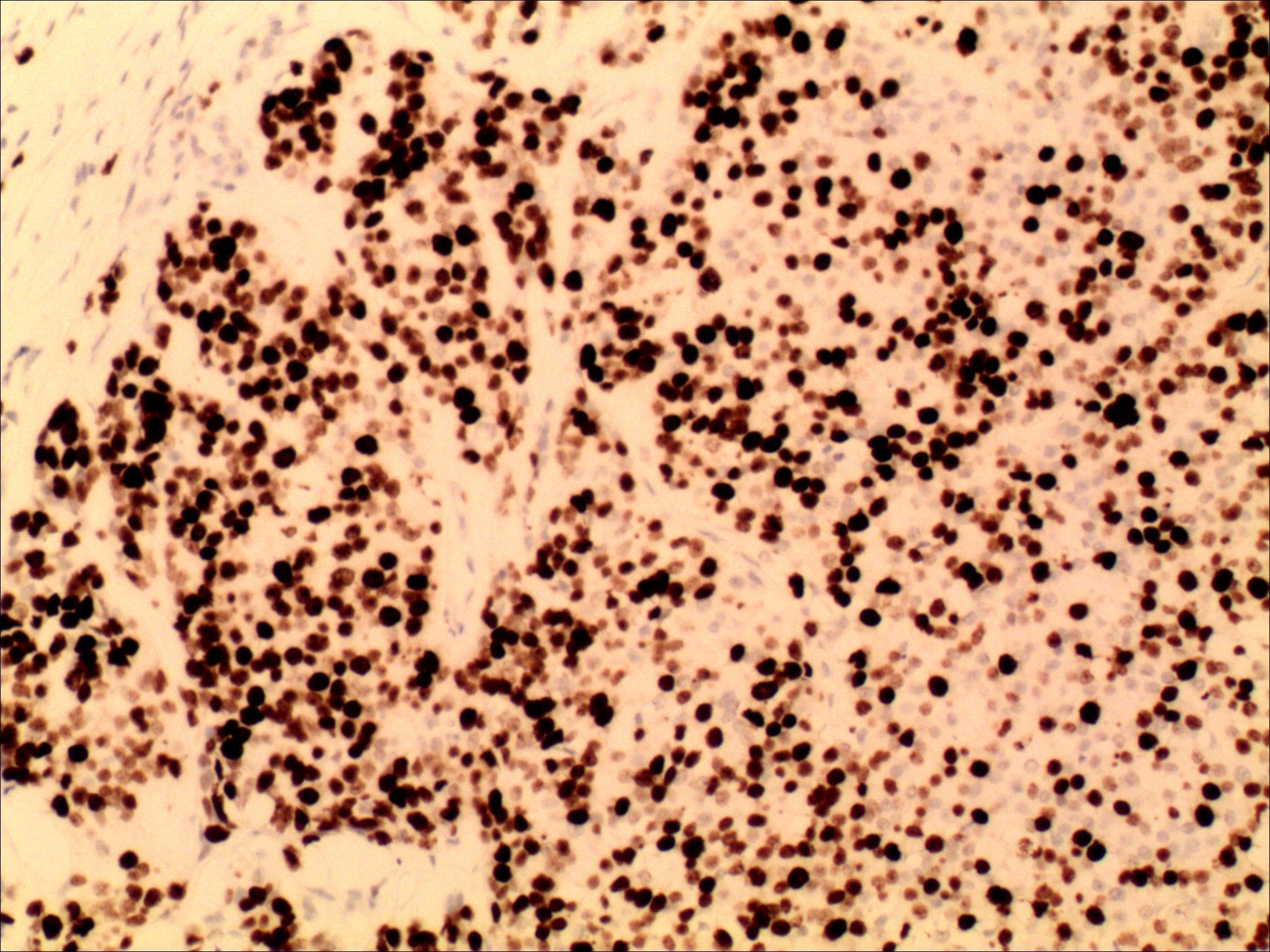

Supplement: Supplementary Figure 6 — Immunohistochemistry: Ki-67 expressed 70%. [file Image6.jpeg]

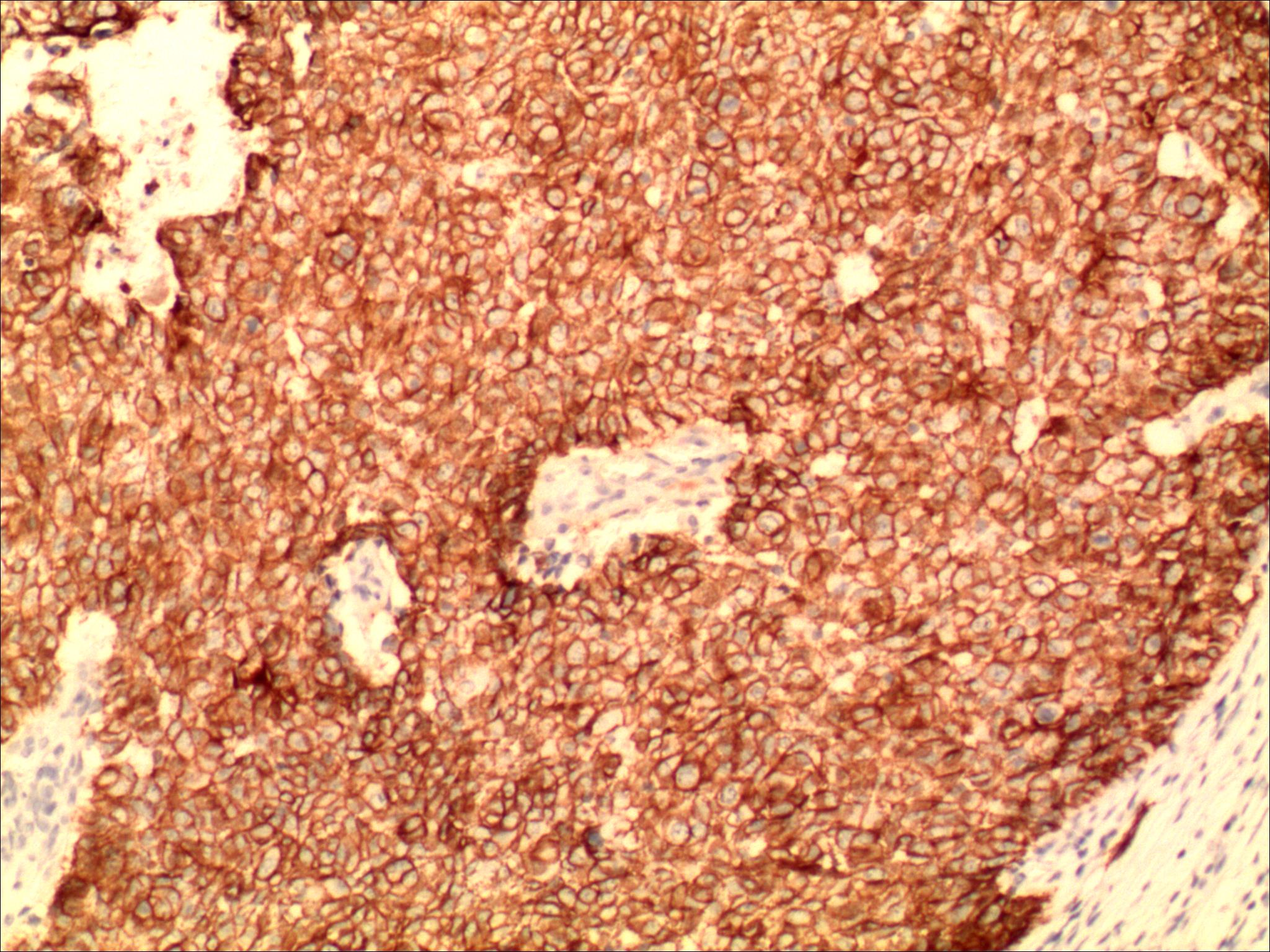

Supplement: Supplementary Figure 7 — Immunohistochemistry: E-Cadherin is positive. [file Image7.jpeg]

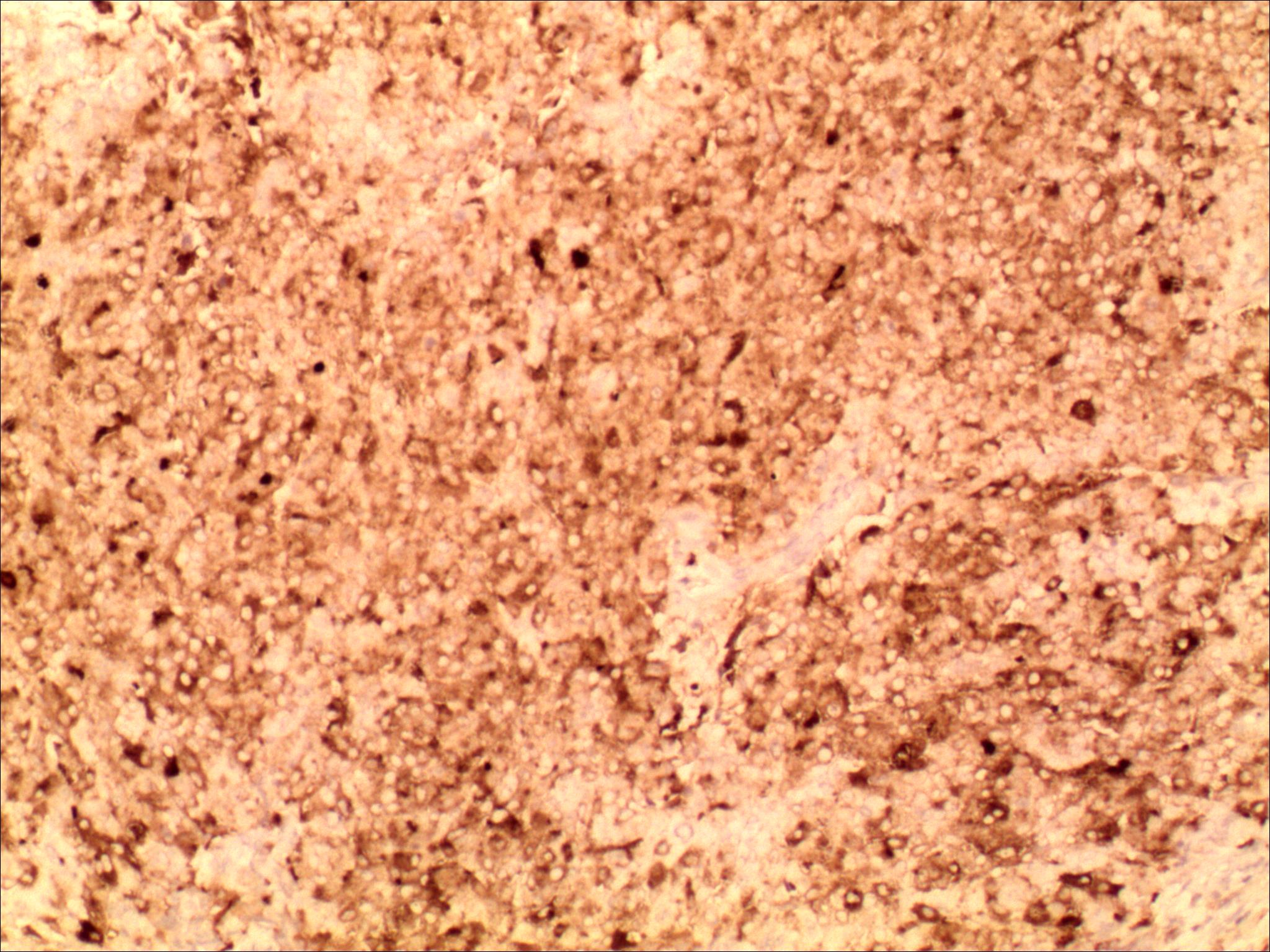

Supplement: Supplementary Figure 8 — Lysozyme is positive, which is a marker of serous differentiation. [file Image8.jpg]
